# Inhibitory Activity of the Flower Buds of *Lonicera japonica* Thunb. against Histamine Production and l-Histidine Decarboxylase in Human Keratinocytes

**DOI:** 10.3390/molecules19068212

**Published:** 2014-06-17

**Authors:** Yoshihiro Inami, Yuko Matsui, Tomoko Hoshino, Chiaki Murayama, Hisayoshi Norimoto

**Affiliations:** 1 Fundamental Research Laboratories, Hoyu Co., Ltd., Roboku 1-12, Nagakute City Aichi 480-1136, Japan; E-Mails: yoshihiro_yinami@hoyu.co.jp (Y.I.); yuko_matsui@hoyu.co.jp (Y.M.); tomoko_hoshino@hoyu.co.jp (T.H.); 2 Kampo Research Laboratories, Kracie Pharma., Ltd., Kanebo machi 3-1, Takaoka City Toyama 933-0856, Japan; E-Mail: murayama_chiaki@phm.kracie.co.jp

**Keywords:** flower buds of *Lonicera japonica* Thunb., chlorogenic acid, histamine, l-histidine decarboxylase, human keratinocytes

## Abstract

In previous studies we found that anionic surfactants such as sodium laurate (SL) and/or sodium dodecylsulfate (SDS) exert actions on epidermal keratinocytes rather than mast cells to give rise of histamine production and skin itching through increasing the expression of the 53-kDa active form of l-histidine decarboxylase (HDC). In addition, with treatment of SL in a three-dimensional human keratinocyte culture, increases in both the 53-kDa HDC and histamine production are detected and thus this culture assay is applied to screen anti-itching materials from natural resources. In this study, the inhibitory activity of “Kin-gin-ka” (flower buds of *Lonicera japonica* Thunb., FLJ) against histamine production and expression of the active form of HDC were examined in this culture assay. FLJ is a well-known traditional Chinese medicine, being used to treat fevers, coughs and some infectious diseases. The result showed both FLJ and chlorogenic acid had inhibitory activities against the expression of 53-kDa HDC and histamine production. However, chlorogenic acid showed a weaker effect on histamine production than that of FLJ, suggesting that other chemical constituents besides chlorogenic acid could contribute to the inhibitory activities. Thus, a further chemical study of FLJ is now under investigation.

## 1. Introduction

Various detergents are used in our daily life is a wide variety of toiletry and household cleaning products. In other words, our skin is frequently exposed to those chemical detergents. In some cases, some of these detergents in shampoos and soaps could cause adverse effects on the skin like irritation, dryness and itching [1,2]. At present, anionic surfactants are used in many these detergents in order to achieve stronger detergency, among of them, sodium laurate (SL) and/or sodium dodecylsulfate (SDS) are reported to cause irritation reactions in human and animal skin [3,4,5].

In our recent studies, we found [6,7] that topical application of 1%‒10% SL or 10% SDS induces itch-related behaviors (scratching) in mice, and they are is suppressed by terfenadine, a H_1_ histamine antagonist. Furthermore, such itch-related behaviors are observed in mast-cell deficient mice treated with SL, and epidermal histamine production is enhanced at the same time, suggesting that SL exerts an action on epidermal histamine release rather than on mast cells. Histamine is known to be derived from the decarboxylation of the amino acid histidine through a reaction catalyzed by the enzyme by l-histidine decarboxylase (HDC). Recently, Ichikawa *et al.* [8] reported that HDC is initially translated in mammalian as a 74-kDa form and then post-translationally processed into a 53-55-kDa form which has stronger activity than the 74-kDa form. In further studies, the effect of SL on HDC processing was examined [6,7], and it was found that SL enhanced the processing of 74-kDa to 53-kDa HDC in the epidermis, which indicates that epidermal histamine plays a crucial role in the development of these scratching behaviors. In addition, both 74-kDa and 53-kDa HDCs are also detected in a three-dimensional human keratinocyte culture in which SL increases the ratio to 53-kDa HDC to 74-kDa HDC and the production of histamine [6]. Thus, this culture assay with three-dimensional human keratinocytes could be a very effective screening tool to find novel materials against skin itching and/or as model of skin irritation test to replace animal testing because animal tests in cosmetics have been currently prohibited in the European market [9]. 

In this study, the inhibition of a water extract of crude drug “Kin-gin-ka” (flower buds of *Lonicera japonica* Thunb., FLJ) and its main chemical constituent chlorogenic acid against SL-enhanced histamine production and HDC in human keratinocytes were examined in detail on the basis of the results of a pre-screening assay in the course of a search for anti-itching materials from natural medicines. “Kin-gin-ka” is one of the most widely used traditional Chinese medicines for treatment of exo-pathogenetic wind-heat, and some infectious diseases [10], and it possesses various pharmacological actions such as against microorganisms, oxidation and inflammation [11,12,13].

## 2. Results and Discussion

### 2.1. HPLC Analysis of FLJ Extract

**Figure 1:**
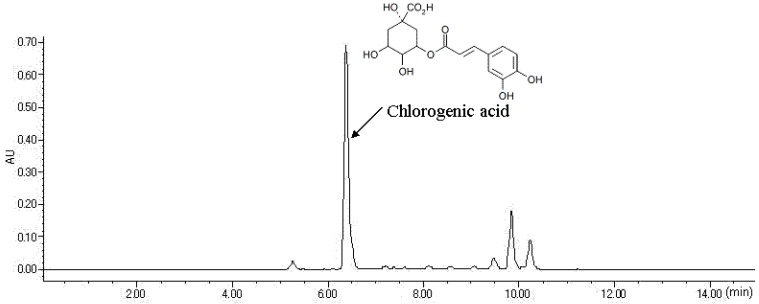
HPLC profile of “Kin-gin-ka” (flower buds of *Lonicera japonica* Thunb.) extract. Contents of chlorogenic acid was calculated on the basis of a standard curve (y = 13122194.8x + 1709916, r^2^ = 0.9902) within a certain concentration range (0.021–2.10 mg/mL).

As shown in Figure 1, the HPLC profile indicated that chlorogenic acid (Rt: 6.38 min) was the major chemical constituent of FLJ and its contents was 63 mg/g of extract powder in the light of absolute quantitative chemical analysis.

### 2.2. Effects of the FLJ Extract and Chlorogenic Acid on SL-Enhanced Histamine Production

**Figure 2:**
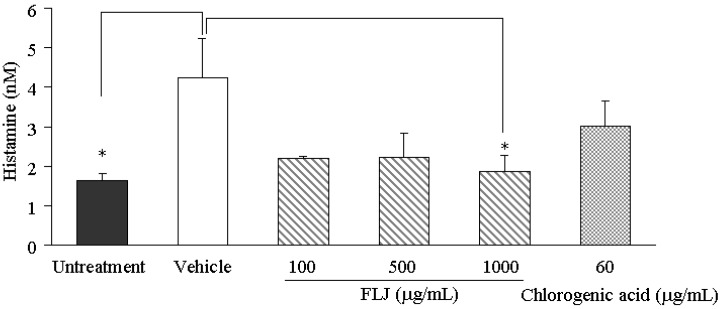
Effects of the FLJ extract and chlorogenic acid on the concentration of histamine enhanced by SL treatment in a three-dimensional human keratinocyte culture. Values represent the means ± SEM (*n* = 3). * *p* < 0.05 *vs.* Vehicle treated with distilled water (Dunnett’s test).

After treatment with 100–1,000 µg/mL of FLJ extract (corresponding to a chlorogenic acid concentration of 6.3–63 µg/mL), the SL-enhanced histamine production was suppressed in the three-dimensional human keratinocyte culture in a non-concentration dependent manner although at the concentration of 1,000 µg/mL it was significant (Figure 2). Chlorogenic acid (60 µg/mL), however, showed a weaker inhibition than that of FLJ that it was determined on the basis of its contents in FLJ at a concentration of 1,000 µg/mL. In addition, no effect on cell viability was observed with ratios to vehicle of 107.5%, 102.2%, 101.8% and 102.3% in each group, respectively (data not shown). This result suggested that other chemical constituents could contribute to the inhibitory activity against SL-induced histamine inhibition of FLJ besides chlorogenic acid. Hence, further chemical study is necessary to clarify this. 

It should be noted that chlorogenic acid significantly inhibited SL-induced histamine production in murine epidermis [14], however, in the present study, such a significant inhibition was not indicated. Although the reason is unclear, it may be speculated that chlorogenic acid has an effect on the histamine production in other cells such as mast cells in addition to keratinocytes in the epidermis. 

### 2.3. Effects of the FLJ Extract and Chlorogenic Acid on the Expression of HDC Induced by SL Treatment

**Figure 3:**
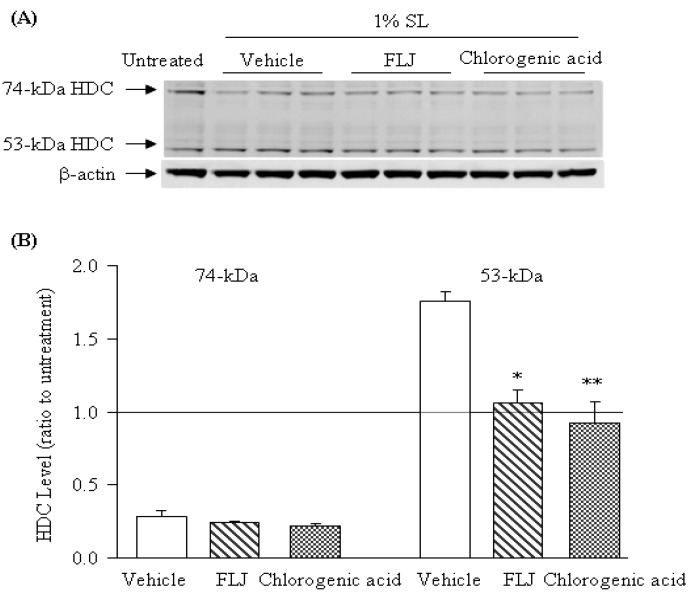
Effects of the FLJ extract (1,000 µg/mL) and chlorogenic acid (60 µg/mL) on the HDC expression of the three-dimensional human keratinocyte treated with 1% SL. (**A**) Detected by western blotting analysis; (**B**) The β-actin levels were evaluated as a loading control, and the data are expressed as the 74-kDa or 53-kDa HDC/β-actin ratio. A solid line denotes the naive level (untreated). Values represent the means ± SEM (*n* = 3). * *p* < 0.05, ** *p* < 0.01 *vs.* Vehicle treated with distilled water (Bonferroni’s test).

As shown in Figure 3, the effects of both FLJ extract (1,000 µg/mL) and chlorogenic acid (60 µg/mL) on HDC expression were further examined in the three-dimensional human keratinocyte culture after treatment with 1% SL, and they did not suppress 74-kDa HDC expression levels compared to the vehicle. 

On the other hand, they significantly decreased the expression of the active 53-kDa HDC form with almost the same potency. This result is consistent with our previous findings that chlorogenic acid inhibits SL-enhanced HDC processing in the murine epidermis [14]. Nitta *et al.* [15] reported that chlorogenic acid inhibits recombinant human HDC at 10 mM. Taken together, these findings suggested that chlorogenic acid may suppress histamine release of keratinocytes by either inhibiting the expression of 53-kDa HDC or HDC inhibition. 

## 3. Experimental Section

### 3.1. Sample Preparation and HPLC Analysis

Dried crude FLJ drug (No. H121012220, collected in Henan Province, China) was provided and identified by Kracie Pharma., Ltd. (Takaoka, Japan). Approximately 240 g of FLJ was pulverized and extracted with distilled water (1,920 mL) under reflux for 30 min, and then the extract was lyophilized to obtain extract with a yield of 18.6%. The voucher specimens are deposited at the herbarium of Kracie Pharma., Ltd. For HPLC analysis (injection 10 µL) of the FLJ extract was performed with a model 2690 HPLC system equipped with a model 996 photodiode array detector (Nihon Waters Co., Ltd., Osaka, Japan) at 340 nm and a Capcell Pak C_18_ UG120 column (4.6 mm i.d. × 250 mm, particle size 5 μm, Shiseido Co. Ltd., Tokyo, Japan). The column was eluted with acetonitrile in 0.1% phosphoric acid using 10%–100% linear-gradient for 30 min at 40 °C with flow rate at 1.0 mL/min. Chlorogenic acid (purity > 95%, Cayman Chemical Co., Ann Arbor, MI, USA) was dissolved in 50% ethanol before use as was the FLJ extract.

### 3.2. Three-Dimensional Keratinocyte Culture and Treatment Methods

The Labcyte Epi-Model system (Japan Tissue Engineering Co., Ltd., Gamagori, Japan) is a three-dimensional keratinocyte culture, of which prepared from normal human keratinocytes obtained from neonate foreskin with free of other epidermal cells such as melanocytes. SL (Nacalai Tesque, Inc., Kyoto, Japan), chlorogenic acid, and the powder of FLJ extract were dissolved in distilled water. SL solution (1% w/v, 100 µL) was applied to the keratinous layer of the culture for 1 min and then immediately removed. The external surface of the culture was washed three times with distilled water and then left to stand in 5% CO_2_ and air at 37 °C for 2 h, and thereafter treated with 200 μL of chlorogenic acid or FLJ extract for 30 min.

### 3.3. Western Blotting

Protein was extracted from the Labcyte Epi-Model system using a mammalian cell lysis kit (Sigma Aldrich,St. Louis, MO, USA), and concentration of protein measured using a 2-D Quant kit (GE Healthcare Bio-Sciences Corp., Piscataway, NJ, USA). Protein extracts (15 μg) were electrophoresed on a NuPAGE^®^ 4%–12% Bis-Tris gel (Invitrogen Co., Carlsbad, CA, USA) and transferred to a polyvinylidene difluoride membrane. Concentration of protein measured using a 2-D Quant kit (GE Healthcare Bio-Sciences Corp.). After blocking with 1% skim milk in PBS containing 0.1% Tween 20, the membrane was cut, at approximately 50-kDa point, and the upper and lower parts were reacted overnight with rabbit polyclonal anti-HDC (Progen Biotechnik GmbH, Heidelberg, Germany) and anti-β-actin antibodies (1/1,000 each, Abcam, Tokyo, Japan) at 4 °C, respectively. After washing with PBS containing 0.1% Tween 20, the membranes were incubated with fluorophore-labeled donkey anti-rabbit IgG antibody (1/1,000) for 2 h at room temperature. These membranes were then scanned using a fluorescence scanner (Typhoon; GE Healthcare, Munich, Germany) and positive bands were quantified using Scion Image (Scion Corp., Frederick, MD, USA).

### 3.4. Enzyme Immunoassay for Histamine Production

For the determination of histamine production, the three-dimensional human keratinocyte culture was placed on 500 μL of culture medium and maintained in an incubator in 5% CO_2_ and air at 37 °C. Two hours after treatment with 1% SL, the culture medium and keratinocyte culture were collected and then used for the assay. The keratinocyte culture was homogenized in 300 μL of a mammalian cell lysis buffer (Sigma) using a Precellys 24 tissue homogenizer (Bertin Technologies, Montigny, France), and centrifuged at 10,000 × *g* for 10 min at 4 °C. The concentration of histamine in their supernatant and culture medium were determined using a histamine enzyme immunoassay kit (Immunotech, Marseilles, France). 

### 3.5. Statistical Analysis

The data are presented as means ± SEM. Statistical differences were analyzed by Dunnett’s test or Bonferroni’s test using StatLight software (Yukms Co., Ltd., Tokyo, Japan). Values of *p* less than 0.05 were accepted as statistically significant.

## 4. Conclusions

In the previous studies, we found anionic surfactants such as sodium laurate (SL) and/or sodium dodecly sulfate (SDS) exert actions on epidermal keratinocytes rather than mast cells to give rise to histamine production and skin itching by increasing the expression of the 53-kDa active form of l-histidine decarboxylase (HDC). In addition, with treatment of SL in a three-dimensional human keratinocyte culture, both increases in the 53-kDa HDC and histamine production are detected and thus this culture assay is applied to screen anti-itching materials from natural resources. [6,7]. In this study, the inhibitory activity of “Kin-gin-ka” (flower buds of Lonicera japonica Thunb., FLJ) against histamine production and l-histidine decarboxylase were examined in this culture assay.

The result showed both FLJ and its main chemical constituent chlorogenic acid had inhibitory activities against the expression of 53-kDa HDC and histamine production. However, chlorogenic acid showed a weaker effect on histamine production than that of FLJ, and suggesting other chemical constituents besides chlorogenic acid could contribute to the inhibitory activities. Thus, a further chemical study of FLJ is in progress.
